# Mouse Oocytes and Embryos Cryotop-vitrification Using Low Concentrated Solutions: Effects on Meiotic Spindle, Genetic Material Array and Developmental Ability

**Published:** 2013-04

**Authors:** Sahar Almasi turk, Amrollah Roozbehi

**Affiliations:** 1 Anatomy and Cell Biology Department, Bushehr University of Medical Sciences and Health Services, Bushehr, Iran; 2 Molecular and Cellular Biology Research Centre, Shaheed Beheshti University of Medical Sciences and Health Services, Tehran, Iran; 3 Anatomy and Cell Biology Department, Yasuj University of Medical Sciences and Health Services, Yasuj, Iran

**Keywords:** Cryotop, Dimethylsulphoxide, Embryo, Ethyleneglycol, Immunocytochemistry, Mouse, Oocytes, Vitrification

## Abstract

***Objective(s):*** The examination of the possibility of applying lower CPA- concentrations and obtaining the similar results to those using higher concentrations; as it is shown, the toxicity of the CPAs used in vitrification approach will diminish.

***Materials and Methods:*** Following vitrification/warming, oocytes were subjected to PZD/ICSI. SRs, FRs, and DRs were recorded. SRs and DRs of the embryos were monitored after vitrification/warming. IHC studies were done. Data were analyzed in comparison to the data of Exp. (experimental groups) applying 1.5 M CPA- concentrations (largely-used concentration).

***Results:*** The data of oocytes exposed to 1.25 M concentrated CPAs were in consistency with those exposed to 1.5 M and fresh oocytes in terms of SRs, FRs and DRs. Normal spindle and chromatin configuration is in consistence between the two experimental groups, but lower in comparison with control group. The lower the concentrations were, the less SRs, FRs, DRs were. Also, spindle organizations were more normal in comparison with the experimental groups as the concentrations decreased. The results of DRs for embryos which were exposed to 1.25 and 1.0 M concentrated CPAs were close to those vitrified with 1.5 M and fresh embryos but IHC observations in the three Exp. were significantly lower than those of fresh embryos. The results of 7.5 M concentrated CPAs solutions were significantly lower than those of the control group 1.5, 1.25 and 1.0 M treated.

***Conclusions:*** Vitrification by cryotop technology using minimal volume approach increases both cooling and warming rates, therefore, the CPAs limited reduction to 1.25 and 1.0 M instead of using 1.5 M for oocytes and embryos cryotop-vitrification procedure, may be a slight adjustment.

## Introduction

The risk of multiple pregnancies often presents in IVF programs and the existence of factors in stimulated uterine cycle which may jeopardize implantation, are important forces in perfecting embryo cryopreservation. On the other hand, the ethical restriction and assurance of potential fertility following chemo/radio therapy in neoplastic pathologic condition have led scientists to focus on female gamete preservation (1). During ultrarapid freezing (Vitrification), the liquid is so rapidly cooled that it forms into a glassy, vitrified solid state from the liquid phase at low temperature, not by ice crystallization, but by extreme elevation in viscosity during cooling (1). Basically, vitrification approach totally eliminates one source of chilling injury, ice crystal formation, and avoids zona fracture (2); however, it exposes cells to a considerably elevated toxic and osmotic effect (3). 

The normal physiology of the freezed-oocytes and embryos is detected by successful developmental consequences which in turn is dependent on having normal spindle apparatus and genetic material array in cells. The probable cause of reduced viability of cell during freezing and thawing is the disruption of cell organelles, particularly the CSK organization and genetic material array, as a result of intracellular ice formation (4). On the other hand, preserving freezed-gametes and qualified embryos until the time that they need to be used, is fundamental in all cryostorage methods (5). Researchers consider the integrity of the CSK structure and cell functionality as a valuable parameter for the quality of frozen-thawed cells (6). Obviously, decreasing CPA- concentrations and therefore the toxicity would be a step toward the higher security level of the cryopreservation technique (7). 

Researchers have introduced various cryopreserving methods and protocols through changing freezing solutions, introducing new different cryocontainer and examining various cooling/warming rates all over the world. Above all, preserving freezed-gametes and qualified embryos until they need to be used is fundamental in all cryostorage methods. Would it be possible to decrease cryoprotectants concentration and therefore the toxicity, in order to develop higher security cryopreservation technique?

In the present study, through investigating developmental consequences, spindle apparatus morphology as well as the genetic material array of cryotop-vitrified mouse M-II oocytes and four cell stage-embryos using low concentrated CPAs solutions, we re-examine the vitrification protocol and improve the technique.

## Materials and Methods

This was an experimental study. Animals were taken care of according to the university guide for the care and the use of laboratory animals, including 12/12 light/dark, 18-22°C and fed based on a diet containing straw and barley grains. All chemicals were purchased from Sigma unless otherwise stated. 

*Reagents and media*

The Vitrification kit was purchased from Kitazato Biopharma, Mitojima, Japan. The medium for oocytes manipulation and embryos culture was Hypermedium, as Eroglu *et al *indicated (8). Before use, the drops of the Hypermedium were overlaid with embryo-tested mineral oil and equilibrated overnight under a humidified atmosphere of 6% CO_2_ in air at 37^o^C. 

*Egg and embryo collection*

Oocytes and embryos were obtained from 8 to 10 wk-old C57BL/6J mice; Pasteur Institute animal house, Iran. Superovulation was induced as described previously (9). M-II oocytes and four cell stage-embryos collecting techniques were detailed elsewhere (7). 

*Vitrification/warming*

M-II oocytes and four cell stage-embryos were vitrified/ warmed by the minimum volume cooling method using cryotop as exactly as mentioned by Kyono *et al *(10). There were five or six oocytes/embryos on each cryotop. The oocytes/embryos with a poor grade, irregular contours, dark coloration, or fragmented unequal blastomeres were excluded from cryopreservation. Cryostorage was done in LN2 for 5 days. Re-expanded oocytes/embryos were considered to have survived and transferred to the Hypermedium for recovery before experimentation.

*The fertilization and development of cryopreserved oocytes*

Sperm preparation was performed as previously described (11). Oocytes PZD and ICSI were performed after 1 h-incubation period following the method of Balaban *et al *with brief modifications (12). The zona pellucida of cryosurvived oocytes (assessed by the translucent appearance of cytoplasm, the integrity of the plasma membrane and the zona pellucida, the size of the perivitelline space and extruded polar body) was dissected on 10–15% of its circumference with a fine glass needle, far from the polar body area at 37^o^C. Morphologically normal, motile spermatozoa were randomly selected for ICSI. Separated sperm heads were injected into the PZD-oocytes at 17^o^C on a pre-cooled inverted microscope. Injected oocytes were left to rest for 20 min. at 17^o^C followed by 15 min. at room temperature (RT). All oocytes after ICSI were washed four times in Hypermedium 0.4% BSA (w/v). Fertilized oocytes were cultured in a 60 mm tissue culture dish in 20 µl of Hypermedium ‏0.4% BSA (w/v) under mineral oil. The presumptive zygotes were developed to two cell-stage embryos, which were scored 6 and 24 hr after insemination, respectively.

The culture of cryopreserved embryos

Cryosurvived embryos (assessed by morphologically normal blastomeres with apparent zona pellucida integrity) were cultured in a 60 mm tissue culture dish in 20 µl of Hypermedium ‏0.4% BSA (w/v) under mineral oil with incubation at 37^o^C under a 6% CO_2_ atmosphere. Within a few hr, the cryosurvival of the embryos was assessed by their morphological appearance. Then, the developmental competence of the embryos was evaluated by their ability to develop to 8-cell, morulla, young blastocyst and hatching/hatched-stage blastocyst after 48, 72, 96, 100-120 h in culture medium, respectively.

Spindle and chromatin visualization

Spindle and chromatin configuration were assessed using the fluorescent methods as follows: a number of both treated and fresh oocytes/embryos immediately after warming and the other ones after one hr-incubation period, were fixed in prewarmed (37^°^^C^) 2% PFA in DPBS with 0.04% Triton X-100 for 1 hr. They were then washed in washing buffer DPBS containing 0.1% BSA (w/v) two times (15 min. each) and left in Triton X-100 0.1% solution for 30 min. for permeabilization. Non-specific binding was blocked using goat serum 10% for 1 hr. To spindle/chromatin visualization, fixed oocytes/embryos were incubated in mouse monoclonal anti-α-tubulin (1/100) in DPBS with 0.05% BSA (w/v) for 1 hr at 37°C. Oocytes/embryos were then washed and stained with FITC conjugated to goat anti-mouse (1/150) in DPBS + 0.05% BSA (w/v) for 1 hr in the dark. After the sec wash (30 min. each at RT), samples were stained for chromatin with DAPI (1/1000) in DPBS + 0.05% BSA (w/v) for 30 min, in the darkness at RT, mounted on glass slides. The localization of meiotic spindle and chromatin revealed by FITC and DAPI fluorescence using a fluorescence microscope (Leica DMIL; Germany) with optical filters specific for the wavelengths of 450-490 nm and 330-380 nm to detect the green signal of FITC and the blue signal of DAPI.

**Figure 1:**
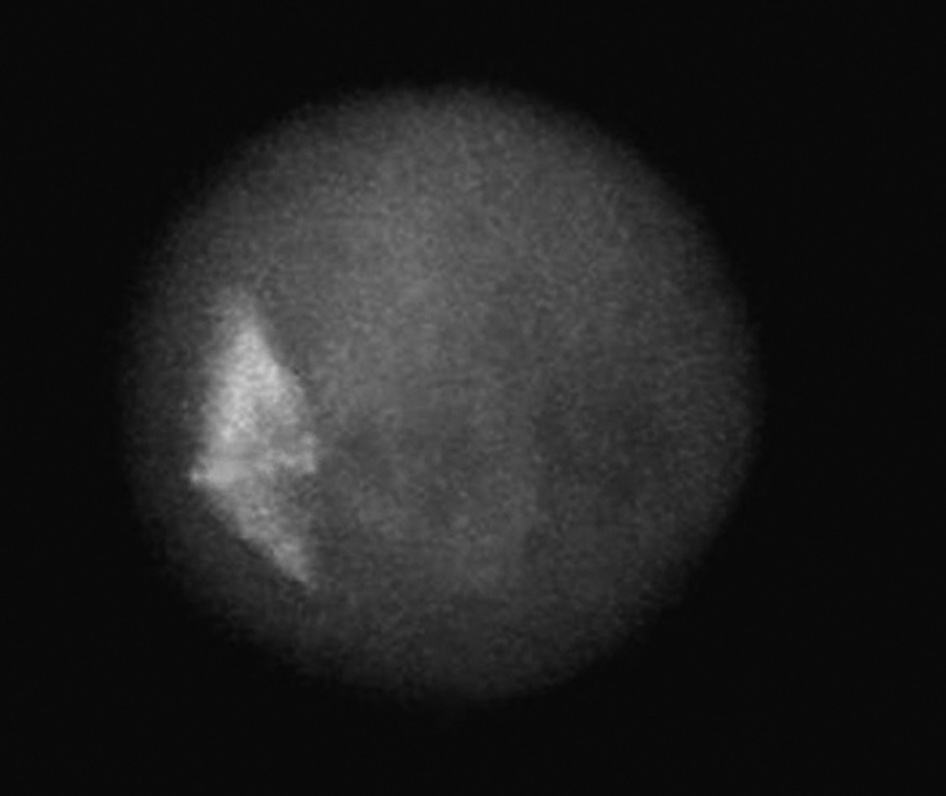
The fluorescent micrograph of control oocyte shows the examples of normal spindle and chromatin configurations. A fresh oocyte stained immunocytochemistically with anti α-tubulin monoclonal antibody and fluorescein isothiocyanate to visualize the spindle (green), along with counterstained with Diamidino-2-phenylindole to visualize the chromosomes (blue). After freezing at the metaphase-II stage and warming, the subcortically localized normal spindle configuration appears barrel-shaped with chromosomes equatorially arranged on a regular plate

**Figure 2:**
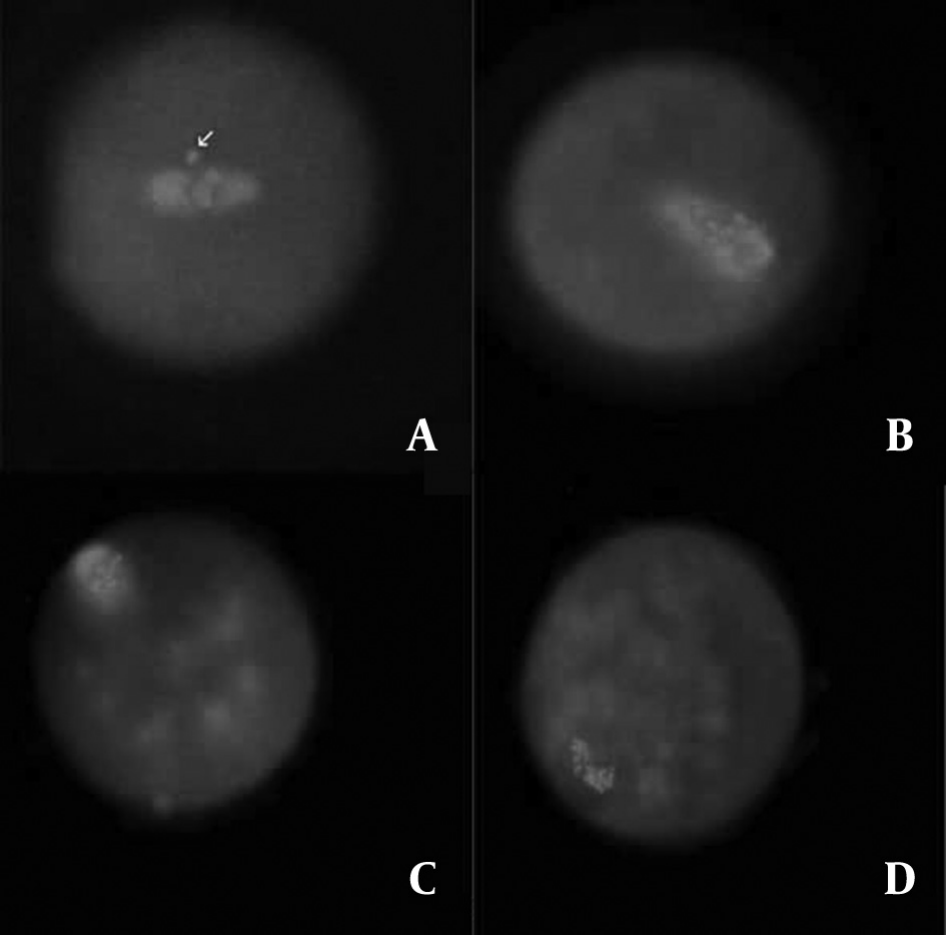
The fluorescent micrographs of the treated oocytes show the examples of spindle and chromatin configurations abnormalities. Spindles were stained in green and chromosomes in blue. Arrow in panel A indicates chromosomes displaced out of the abnormally localized spindle. In panels B and C, in addition to abnormal localization, the spindles appear not to have distinct poles. In panel B, in spite of one compaction, various compact masses of chromatin exist. In panel D, in contrast to complete depolymerization of spindle microtubules, it seems chromosomes did not become dispersed. Rather, they remained close together in disorganized bundles

Morphologically normal meiotic spindles and chromosome alignments in oocytes were barrel-shaped with two distinct poles, and metaphase plate-aligned compact group, respectively (Figure 1). Any other configurations were considered as abnormal (Figure 2).

**Figure 3:**
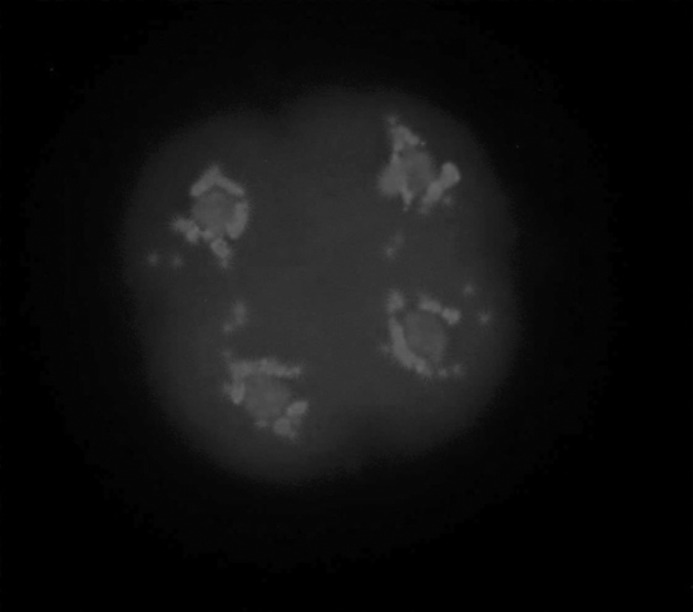
The fluorescent micrograph of control embryo shows the examples of normal genetic material array. A fresh embryo stained immunohistochemically with anti α-tubulin monoclonal antibody and Fluorescein Isothiocyanate to visualize the microtubules (green) and counterstained with Diamidino-2-phenylindole to visualize the chromosomes (blue). After freezing at the four cell stage-embryo and warming, four compact masses of chromatin, each in one blastomere are considered normal.

**Figure 4:**
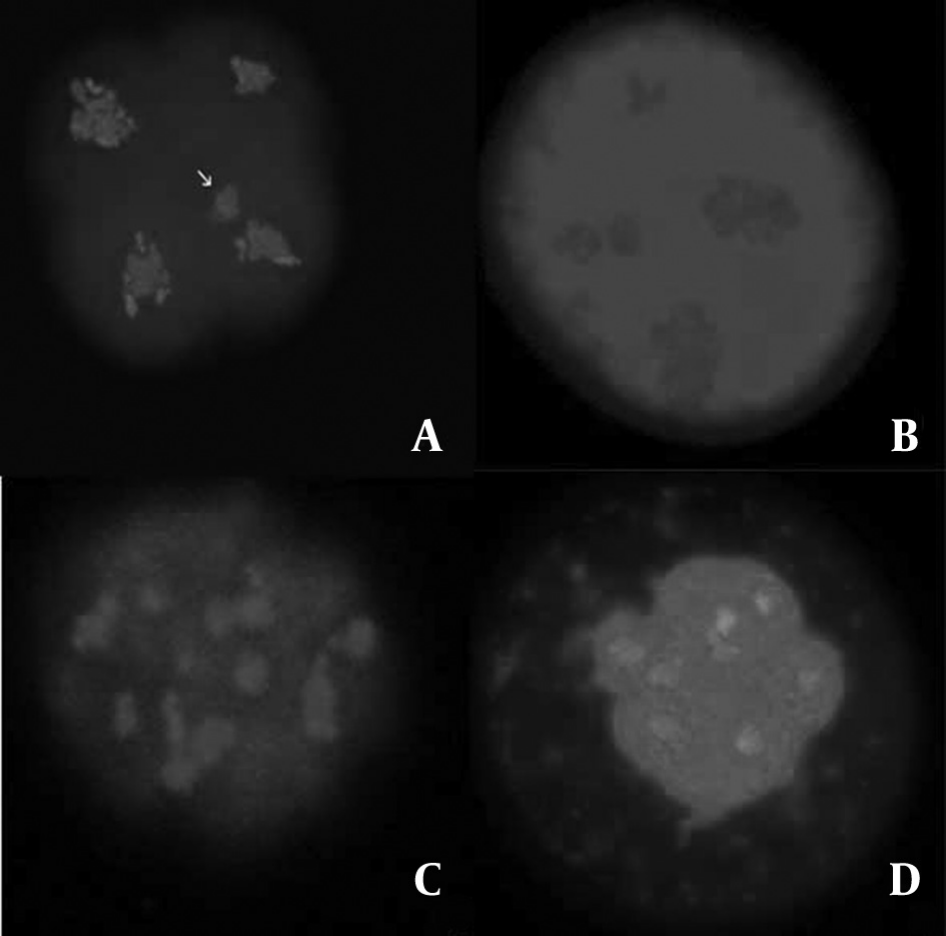
The fluorescent micrographs of treated embryos show the examples of chromatin configurations abnormalities. Microtubules were stained in green and chromatin in blue. Arrow in panel A indicates a separate part of chromatin displaced away from the rest of mass in one blastomere. The embryo in panel B seems to have unusually large and the small masses of chromatin in different blastomeres. The embryos in panel C and D show the several compact masses of chromatin, atypical in size and distribution**.**

The evidences of the morphologically normal chromatin arrangement in vitrified embryos, in comparison with the images of fresh embryos were observed, in a way that the existence of four compact masses of chromatin, each in one blastomere (Figure 3) was considered normal and any other configurations were considered abnormal (Figure 4). Furthermore, according to the method described by Zenzes *et al *(13), the amount of fluorescent MTs in treated embryos being as near as possible to those of the control embryos (Table 4) provides explanation to consider MTs in vitrified embryos morphologically normal.

Experimental design

M-II oocytes and four cell stage-embryos were allotted randomly to one of the following control and five experimental groups (Exp.): The oocytes of Exp. 1 to 5 were subjected to PZD/ICSI procedures and in vitro culture after vitrification/warming, while 0.75 M ES and 1.5 M VS, 0.625 M ES and 1.25 M VS, 0.5 M ES and 1.0 M VS, 0.75 M ES and 0.75 M VS and 0.375 M ES and 0.75 M VS, were used in order. Similarly, vitrified embryos assigned to Exp. 1 to 5, were cultured in vitro after warming. Non-vitrified fresh oocytes and embryos were considered as control group. Experiments in all series were repeated at least seven times to evaluate developmental ability and four times to assess immunostained organelles.

Statistical analysis

The mean percentage of the differences in the rates of survival, fertilization, further development, having normal spindle apparatus and genetic material array between the control and treatment groups were tested for significance through one-way ANOVA and LSD as post hoc test. The level of significance was set at less than 0.05.

**Table 1:** Metaphase II-oocytes survival, fertilization and developmental rates after using different concentrations of the cryoprotectants

Groups	No. of vitrified oocytes	No. of recovered oocytes	No. of morphologically survived oocytes after warming	No. of inseminated oocytes	No. of fertilized oocytes	No. of developed zygotes to 2 cell
Control	—	—	—	138	122 (88.9±5.2)	115 (94.4±3.9)
Exp. 1	106	103	97 (94.3±5.4)	97	84 (85.6±11.11)	76 (90.3±7.2)
Exp. 2	145	141	127 (90.3±7.3)	127	110 (87.3±8.51)	97 (88.6±8.1)
Exp. 3	140	137	98 (72.4±10.9)*	98	37 (38.1±6.42)*	21 (58.5±17.4)*
Exp. 4	157	151	33 (21.5±4.0)*	33	0*	0*
Exp. 5	187	186	0*	0	0*	0*

Data presented in parentheses as mean ± standard division

* P<0.001

## Results

*Developmental competence*

Oocytes in Exp. 1 (larger-used CPA- concentrations ) showed a statistically non-significant difference in SR with Exp. 2 and significant higher SR with the other Exp. (*P<*0.001). Comparing the results of Exp. 3, 4 and 5 revealed statistically significant decreased SRs in proportion to using lower CPA- concentrations (*P<*0.001). In other words, the less CPAs concentrated solutions were used, the less SRs were obtained. When we compared FRs, the control group and Exp. 1 and 2 exhibited no difference, although FRs in Exp. 1 and 2 were lower than fresh oocytes. The FR in Exp. 3 was significantly lower than those recorded for fresh and Exp. 1 and 2 oocytes (*P<*0.001). None of cryosurvived oocytes in Exp. 4 were fertilized. The DRs to two cell-stage embryo were not different between the control group and Exp. 1 and 2 oocytes. DR to two cell-stage embryo in Exp. 3 was significantly lower than the control group and Exp. 1 and 2 (*P<*0.001). Table 1 provides the details of our observations after using different freezing solutions in order to vitrify mouse mature oocytes.

Embryos in Exp. 1 showed a statistically non-significant different SRs in Exp. 2 and 3 and significant higher SR in Exp. 4 and 5 (*P<*0.001). Cryosurvived embryos in Exp. 4 showed significant lower SR in Exp. 1, 2 and 3 (*P<*0.001). There were significant differences between embryos SR in Exp. 5 and the rest of Exp. (*P<*0.001). There were no statistical differences between DR to 8 cell-stage embryos of Exp. 1 and Exp.2 compared to the control group, whereas DRs of Exp. 1 and 2 were lower than the control embryos. DR to 8 cell-stage embryo in Exp. 3 was significantly lower compared to the control group (*P<*0.001) and Exp. 1 (*P<*0.001) and Exp. 2 (*P<*0.05). In addition to significantly lower results in comparison to Exp. 3, the description was the same for Exp. 4 (*P<*0.001). None of cryosurvived embryos in Exp. 5 were developed further. The rates of embryos developed to morulla were lower, but not-statistically different in control group and Exp. 1, 2 and 3. DR to morulla in Exp. 4 was significantly lower than that recorded for fresh and Exp. 1 (*P<*0.05), whereas, there was no significant difference compared to Exp. 2 and 3. The rates of embryos developed to young blastocyst-stage in all the vitrified groups were lower than those of the control group, however, the difference between the control group and Exp. 1 and Exp. 2 were not statistically significant. DR to young blastocyst in Exp. 3 and 4, were significantly lower compared to the control group, Exp. 1 and 2 (*P<*0.001). The rates of embryos developed to hatching/hatched blastocyst-stage in all the vitrified groups were lower than those of the control group; however, the difference between the control group and Exp. 1, 2 and 3 were not statistically significant. DR to hatching/hatched blastocyst in Exp. 4 was significantly lower compared to the control and vitrified groups (*P<*0.001). Table 2 summarizes DRs of fresh versus vitrified embryos.

*Immunostaining observations*

Not all fresh oocytes showed normal spindle and chromatin configuration immediately after warming; but the number of fresh oocytes with normal spindle apparatus after 1 hr-incubation period was larger. There was no significant difference between these two records at various times after warming (*P<*0.001). This was in contrast with the number of oocytes with normal spindle organization in Exp. 1, 2, 3 and 4 at two different times, which displayed significant differences (*P<*0.001). When comparing the results of Exp. 1 and 2 oocytes, it was noted that the number of oocytes with normal spindle organization both immediately and after 1 hr were similar but significantly lower than those of control oocytes (*P<*0.001). The proportion of oocytes with normal spindle and chromatin configuration in Exp. 3 was significantly lower than the control group, Exp. 1 and 2 (*P<*0.001) at both of the different times. While there were no oocytes with normal spindle apparatus immediately post-warming in Exp. 4, the proportion of oocytes with normal spindle status after 1 h-incubation period was larger, although they were significantly lower than the control group, Exp. 1, 2 and 3. The oocytes in Exp. 5 were detected with normal spindle apparatus neither immediately after warming, nor following 1 hr incubation period. Table 3 shows the details of our observations at different times after warming, and after using different freezing solutions in order to vitrify mouse mature oocytes.

**Table 2:** Four cell stage-embryos developmental rates after using different concentrations of the cryoprotectants

Groups	No. of vitrified embryos	No. of recovered embryos	No. of morphologically survived embryos after warming	No. of cultured embryos	No. of developed embryos developed to 8 cell	No. of developed embryos to morula	No. of developed embryos to young blastocyst	No. of developed embryos to hatching/hatched blastocyst
Control	—	—	—	141	135 (95.9±3.6)	128 (95.0±5.6)	126 (98.4±2.9)	113 (89.9±5.33)
Exp. 1	142	141	134 (95.1±4.7)	134	125 (93.3±3.8)	118 (94.4±4.0)	115 (97.6±4.7)	95 (82.4±5.7)
Exp. 2	147	145	135 (93.3±6.1)	135	125 (92.5±4.1)	116 (92.9±5.3)	112 (96.7±4.8)\	91 (81.4±8.0)
Exp. 3	150	148	136 (91.9±4.8)	136	117 (86.2±7.5)*	108 (93.4±5.9)	91 (86.1±9.7)*	74 (78.2±7.9)
Exp. 4	242	239	124 (52.1±5.8)*	124	85 (68.6±10.0)*	74 (87.9±12.7)*	31 (42.3±8.2)*	10 (31.9±30.53)*
Exp. 5	338	333	50 (15.0±3.6)*	50	0*	0*	0*	0*

Data presented in parentheses as mean ± standard division

* P<0.001

**Table 3:** Metaphase II-oocytes normal spindle organization rate immediately after warming and following one hr-incubation period after using different concentrations of the cryoprotectants

Groups	No. of immediately after warming stained oocytes	No. of immediately after warming stained oocytes with normal spindle apparatus	No. of stained oocytes after 1 hr incubation	No. of stained oocytes after 1 hr incubation with normal spindle apparatus
Control	40	33 (83.1±6.8)	46	43 (93.3±4.5)
Exp. 1	57	13 (23.0±5.7)a	54	46 (85.2±4.9)a
Exp. 2	65	14 (21.5±8.8)a	66	56 (84.9±5.2)a
Exp. 3	74	10 (13.3±4.1)a, b, c	76	48 (63.2±7.6)a, b, c
Exp. 4	92	0	94	10 (10.5±2.9)a, b, c, d
Exp. 5	79	0	80	0

Data presented in parentheses as mean ± SD

a Indicates significance for the Control Group vs. Exp. 1, 2, 3 and 4 (P<0.001)

b Indicates significance for Exp. 1 vs. Exp. 3 and 4 (P<0.001)

c Indicates significance for Exp. 2 vs. Exp. 3 and exp. 4 (P<0.001)

d Indicates significance for Exp. 3 vs. Exp. 4 (P<0.001)

**Table 4.** Four cell stage-embryos normal fluorescence index immediately after warming and following one hr-incubation period after using different concentrations of the cryoprotectants

Groups	No. of stained embryos immediately post-warming	Fluorescence a Intensity immediately post-warming 0 1 2 3				Fluorescence b Index immediately post-warming	No. of stained embryos after 1 hr incubation	Fluorescence Intensity after 1 hr incubation 0 1 2 3				Fluorescence Index after 1 hr incubation
Control	38	0	0	8	30	2.7	40	0	0	0	40	3
Exp. 1	46	13	28	5	0	0.8	48	0	0	5	43	2.8
Exp. 2	50	15	30	5	0	0.8	52	0	2	4	46	2.8
Exp. 3	52	19	29	4	0	0.7	56	3	3	3	47	2.6
Exp. 4	60	36	24	0	0	0.4	62	12	9	9	32	1.9
Exp. 5	64	51	13	0	0	0.2	68	34	14	7	13	0.9

a Each figure refers to the number of embryos that were assigned a subjective rating for fluorescence intensity of the MTs: 0= little or no fluorescence; 1= weak; 2= moderate; 3= strong fluorescence.

b The fluorescence index is the value calculated for a group of embryos vitrified with indicated concentrated solutions; the value refers to the number of embryos rated to exhibit a given fluorescence intensity multiplied by the number of embryos with that rating; that quantity is divided by the total number of embryos in that group.

**Table 5:** Four cell stage-embryos normal chromatin configuration rate immediately after warming and following one hr-incubation period after using different concentrations of the cryoprotectants

Groups	No. of stained embryos immediately post-warming	No. of stained embryos with normal genetic material array immediately post-warming	No. of stained embryos after 1 h incubation	No. of stained embryos after 1 h incubation with normal genetic material array
Control	38	33 (86.6±8.2)	40	38 (97.7±4.9)
Exp. 1	46	12 (26±3.6)a	48	42 (88.2±10.9)a
Exp. 2	50	12 (24±3.7)a	52	45 (87.1±9.1)a
Exp. 3	52	12 (22.3±8.1)a	56	47 (85.6±5)a
Exp. 4	60	9 (14.4±5.6)a, b, c, d	62	31 (49.6±7.2)a, b, c, d
Exp. 5	64	3 (4.1±4.6)a, b, c, d, e	68	6 (8.2±6.1)a, b, c, d, e

Data presented in parentheses as mean ± SD

a Indicates significance for Control vs. Exp. 1, 2, 3, 4 and 5 (P<0.001)

b Indicates significance for Exp. 1 vs. Exp. 4 and 5 (P<0.001)

c Indicates significance for Exp. 2 vs. Exp. 4 and 5 (P<0.001)

d Indicates significance for Exp. 3 vs. Exp. 4 and 5 (P<0.001)

e Indicates significance for Exp. 4 vs. Exp. 5 (P<0.001)

There were significant decreases in fluorescence intensity of tubulin in vitrified embryos immediately after warming. As shown in table 4, the fluorescence intensity of tubulin increased following one hr-incubation period in 37^o^C. Even after incubation, the fluorescence indices of tubulin in Exp. 4 and 5 were lower than those of control, Exp. 1, 2 and 3 which were not significantly different.

Obviously time-dependent restorations in genetic material array were apparent in the control and Exp. groups. However, it were statistically significant for Exp. (*P<*0.001) and not significant for the control group. When comparing the results of Exp. 1, 2 and 3 embryos, it was noted that the number of embryos with normal status both immediately and after one hr were similar but significantly lower than those of the control embryos (*P<*0.001). The percentage of normal embryos in Exp. 4 and 5 were significantly lower than the control group, Exp. 1, 2 and 3 (*P<*0.001) at both different times. Table 5 shows the details of our observations.

## Discussion

Although there have been numerous studies on vitrification of mouse oocytes and embryos, the majority of them have used at least 1.5 M concentrated CPAs as freezing solution. The purpose of the experiment described herein was to examine the possibility of the applying lower CPA- concentrations and obtain the similar results to those using higher concentrations. As it is, the toxicity of the CPAs used in oocytes/embryos vitrification approach will be diminished.

In designing the experiment, we considered the earlier findings published by Tucker *et al* (1). The actual cooling rate during vitrification, and therefore, the efficiency, may still vary extremely depending on the device used (1). Regarding to the capability of the new tool, cryotop, to allow for an even smaller volume of vitrification medium (<0.1 µl) to be used and therefore yield quicker cooling and warming rate (23,000^o^C/min and 42,000^o^C/min) (14), it appears logical to assume that it is an adjustment to use CPA agents at lower concentration, while maintaining the necessary concentration to achieve vitrification.

To avoid a degree of uncertainty surrounding the outcome of the IVF procedure and to achieve success to overcome infertility, using the most qualified gametes and embryos plays the central role in the ART program (15). Cryopreservation protocols’ efficiency is evaluated by the fact that how much they are able to preserve the quality of the freezed-gametes or embryos (16). Regarding efficiency, assessing current vitrification protocols is not an exception. Therefore, we decided to investigate one of the qualified preserving indicators, spindle apparatus and chromatin array to focuse on cryoservived oocytes fertility and embryos developmental ability by the immunostaining technique.

The biophysical detail of CPAs and the mechanisms of freezing/thawing rates are beyond the scope of this paper. Briefly, it is noted that CPAs are organic solutes that help to protect cellular organelles during cryopreservation although they may damage the CSK system as they can be toxic and cause disruptive osmotic damage to the cell (17). Novel approaches have been tested to reduce the toxicity of various solutions that are to be used to vitrify oocytes/embryos. One of the candidate CPA agents was EG, which was very effective and less toxic for mouse oocytes vitrification (18). Kartberg *et al *realized that vitrification with DMSO protects embryo membrane integrity better than solutions without DMSO (19). The incorporation of DMSO into an EG containing medium has at least two advantages: firstly, vitrification is facilitated because of the greater glass-forming characteristics of DMSO; secondly, the permeability of each CPA is enhanced in the presence of the other (20). Therefore, we were more attracted by the current mixed vitrification solution. 

Cobo *et al *obtained excellent 96.9 % SR after vitrification through applying the cryotop method and usual CPA- concentrations (1.5 M) to human oocyte (21). Kuwayama *et al *and Katayama *et al* have reported a 91% and 94% SRs, 81% and 90% cleavage rates (CRs), respectively (22-23). Morato *et al *have scored 94.5% SR and 46.1% CR (24). 100% morphologically survived and 93% CRs of human pronuclear stage vitrified embryo are the highest published results so far (14). Above-mentioned were the teams with dramatic improvement in their cryostorage method. In the current study, the SR, FR, and CR of the oocytes, which were subjected to 1.5 M and 1.25 M of CPAs (Exp.1 and 2) and further development of embryos in similar Exp., were near to those findings. This seems to support the claim that using 1.25 M DMSO+EG for vitrification medium containing 0.5 M sucrose and cryotop as cryocontainer, we are able to obtain the findings comparable with largely-used higher concentrations (1.5 M). We can take credit for our claim because of the immunostaining results of Exp. 2 oocytes/embryos following 1 h-incubation period, which were in consistency with those of Exp. 1. Comparing the IHC results of these two Exp. immediately after warming has shown remarkable spindle and chromatin organization anomalies which were effectively repaired after 1 h incubation at 37^o^C, though they were small but significantly lower than those of control group. 

Following fresh oocytes collection, images showed 17% spindle organization anomalies. To find out the underlying cause of these, we came across the report which had explained adverse effects of hormonal stimulation, asynchrony in nuclear and cytoplasmic maturation under in vitro condition and oocyte manipulation in laboratory, on spindle apparatus abnormalities (13). The egg collection protocol, laboratory condition and used culture media for the present experiments were not excluded from the accepted items. Similar findings were obtained from embryonic control group stating adverse effects not only on oocytes but also on embryos.

In addition to centrosomes associated with the meiotic spindle, mouse oocytes contain MTOCs in the cortical cytoplasm (25). Disruption of the meiotic spindle of mouse oocytes can be reversed after rewarming and incubation at 37^◦^^C^ (25). The absence of centrosomes and MTs in the cortical cytoplasm (such as those that occur in mouse oocytes) is believed to be responsible for irreversible damage to the meiotic spindle after cooling of human, bovine and monkey oocytes to low temperatures (25). Chemical and physical stresses have been shown, in several species, to affect the MT structure of the oocyte meiotic spindle with deleterious consequences on chromosomal organization (26). Oocytes incubation following freezing/thawing for specific times relieves physical and chemical stresses and results in higher intracellular homeostasis (27). 

Restored spindle organization rates of all treated oocytes in current study were not alone in agreeing the positive effects of 1 h-incubation period after warming (28-29), but what deserves attention is the differences in the rates of cryosurvived oocytes and those in similar experimental groups which had normal spindle configuration after incubation. Because of the technical variety of these results, we can not firmly conclude, but these findings argue that the oocytes viability after warming is not meant to be all intracellular structures, miotic spindle in particular, in healthy condition. These may evoke some researchers’ emphasis on investigating morphological, ultrastructural and molecular status of vitrified cells as well as what commonly is done by evaluating developmental consequences (26, 28). The positive effects of post-warming incubation period on embryos was revealed by rising fluorescent MTs amount particularly in Exp. 1, 2 and 3, which were close to those of the control group. As rising MTs amount after incubation, genetic material array returned to more normal status in all Exp. Embryos. Results were similar in Exp. 1, 2 and 3 but statistically lower than those of control group. Using low CPA- concentrations of 1.25 M and 1.0 M, results in similar findings of using 1.5 M CPAs at the embryo CSK level. This optimism takes credit from evaluating developmental competence of Exp. 3. The statistics showed small but statistically significant decrease in 8-cell stage (as resuming development after warming and prepare to compact) and young blastocyst stage (as preparing to produce inner cell mass) but the SRs and DRs to progressed stages did not show any significant trends between Exp. 3 and control, Exp. 1 and 2. According to the results of embryos treated by 1.0 M CPAs (Exp.3) and two early studies showing that mouse embryos can be frozen using lower concentrations of CPAs (i.e., 1.0 M DMSO and 1.2 M EG) with good success rates (9), further studies are stimulated. 

More research and study should be conducted to demonstrate the proof-of-principle that mature mouse oocytes and embryos cryopreserved using reduced concentration of CPAs can develop to term. Proving the applicability of any protocols needs the protocols to be tested on preserving other cryosensitive oocytes of mammalian species, stage-dependent sensitive embryos to damage during vitrification and develop the embryos in vitro (20, 30).

The results of developmental competence and spindle organization of Exp. 3 stated that improper CPA-concentration (1.0 M) for ideal oocytes vitrification was adopted. CPA-concentration reduction, lower than 1.25 M (Exp. 4 and 5), breed irreversible effects on spindle apparatus and developmental ability in proportion to CPA concentrations. Although comparing the FRs and the percentages of oocytes with normal spindle apparatus after incubation of the Exp. 1 with the ones for Exp. 2 and 3 did not show significant differences; 63% of oocytes were with normal spindle structure in Exp. 3 in contrast to their fertility limited to 38%. As this data has been detected in lower CPA- concentration condition, appropriate CPA-concentration at freezing and sucrose concentration at warming procedure should be designed to head toward more effective cryopreservation. Mullen *et al *have shown M-II oocytes can maintain a normal spindle structure after exposure to widely range of osmotic conditions (31). On the other hand, developmental potential is reduced by about 50% across all test conditions. CPA and sucrose concentrations equilibration should not expose cells to osmotic changes more than they can tolerate. According to the other investigations (31-33) and the fact that reduction of CPA-concentration preserves oocytes CSK in a safer way (21), there are various organelles and processes that contribute to fertilization that is negatively affected in such a condition. Further studies may be conducted on removing CPAs procedure by lower concentrated sucrose solutions and numerous dilution steps, as per using lower CPA- concentrations as freezing media.

Kim *et al *have noted that MTs and MFs are integrated during fertilization (34). These CSK element interactions are required for the union of sperm and egg nuclei and subsequent cell division. In mature mouse and rat oocytes, MFs are mainly located in the cell cortex overlying the meiotic spindle. This domain, rich in MFs, appears to be responsible for maintenance of the meiotic spindle and chromosomes in a peripheral position. Whenever MF-rich domain is segregated, the spindle and chromatin migrate toward the center of the egg (34). Images were taken from Exp. 3 demonstrating migration of the spindle apparatus from the subcortical area to the center. This, as documented earlier, may be the major cause of the formation of two female pronuclei without polar body extrusion (34), which was considered as fertilization indicator in this study. Our results are in contrast with those presented by Paynter (35), absent or abnormal spindles were observed in all of the oocytes cryopreserved which survived but failed to fertilize. As Isachenko and Nayudu have suggested (36), we set out discrepancies between higher proportions of oocytes with barrel-shaped spindles after freezing and subsequent low FRs suggesting that less obvious defects of spindle/chromatin, which may have not been detected by the staining technique, and oocytes fertilization are also expected to be influenced negatively. From an intracellular structural level, observations of different CSK elements simultaneously after cryopreservation should extend to larger numbers and more refined methods.

Katayama *et al* have put forward an interesting theory of MC in oocytes, backing up their argument thorough data which would be helpful to explain the probable cause of the low fertility potential of oocytes after freezing (37). MCs are essential cell organelles, as well as ATP synthesis, involved in intracellular Ca^2+^ homeostasis, a key ion for normal fertilization to happen. Perinuclear aggregation of MC rapidly facilitates the energy supply at fertilization and early embryonic stages may be positively correlated with the developmental ability of embryos. MC distribution pattern is altered in IVF oocytes. The extreme low temperature using in cryopreservation, is also another non-physiologic condition. Also, there isno physiologic protection against it in cells (38), which may be one of the reasons for low fertilization potential subsequent to the worsening alteration of the MC distribution pattern (39) or the MC structure disordering (28) and the Ca^2+^ homeostasis disturbing (40) in oocytes vitrified by rather low protective level, as conducted in Exp. 3 oocytes.

To focus on contributing causes of the low fertility rates of Exp. 3 oocytes, we should call attention to Ca^2+^ and the main component in Ca^2+^ releasing system, ER. Because Ca^2+^ release is essential for several aspects of successful fertilization, it is critical for vitrified oocytes to preserve the ability to release Ca^2+^ (40). Even if MTs are disrupted and a spindle is able to reform within a few hr after oocyte warming, it is possible that the association between the ER and MTs does not re-establish within this time (40).

Using low CPA-concentrations, the toxicity on CSK will be reduced. Although this has been widely acknowledged, information gained by the fore mentioned studies provide insight into the other intracellular structures and functions that may be compromised by vitrifying using such low concentrations. Their susceptibility to temperature fluctuation more than that of spindle apparatus seems to be a subject of our near future examination.

In consistent with the previous observations by many investigators of oocytes cryopreservation methods (13), the immunofluorescent technique that we used shows degraded displaced spindles, several chromatin masses in spite of unique compact chromatin arrangement. Similar to some other studies (25), we observed) complete depolymerized spindle while chromosomes tend to remain together. This suggests that kinetochores may remain associated with MTOCs (26). Gomes *et al *have mentioned the positive correlation between the incidence of spindle repolymerization after vitrification/ warming and the chromatin cohesiveness during cryopreservation (41). Larger majority of the oocytes in Exp. 1 and 2 in comparison with the other Exp. has displayed cohesive chromatin after 1 h-incubation period, which may validate this eventuality when incubation period longer than 1 h is appropriate to restore spindle organization; this issue is yet to be understood accurately (29). Observing similar images taken from the rest of Exp. showed that low success rates would encourage further examinations using the protocols with longer incubation period post-warming.

Further developments and evidences based on SRs, FRs as well as ICC results of the Exp. 4 and 5, were totally disheartening and oocytes return to physiologic functional status was not confirmed using the protocol. Although the precise nature of the damage caused by cryopreservation remains to be exactly determined (9), findings of several studies suggest that the major obstacles in successful oocytes cryopreserving are the characteristics of the oolema (42), the presence of cortical granules, spindle system at the metaphase of meiosis II (13) and zona pellucida hardening (43). In addition, the oocytes must be fertilized by sperm at the appropriate time (18). 

Intracellular ice formation can be affected by the presence of the CPAs in the freezing solutions, and by the freezing and thawing rate (44). To consider important issues like temperature reduction rate, it should be noted that at cooling rates slower than the optimum rate, cell death is due to long periods of exposure to hypertonic conditions. At cooling rates faster than the optimum, cell death is associated with intracellular ice formation, which is inevitably lethal (45). The actual value of the optimum rate is determined by a number of biophysical factors: 1. Cell volume and surface area 2. Permeability to water 3. Arrhenius activation energy (temperature-dependent energy required for the rate of chemical reactions) 4. Type and concentration of CPA additives (45). The last item establishes a connection between the concentration of the CPAs used and the cooling/warming rate. Being able to successfully adopt the CPA- concentrations used for Exp. 4 and 5, a new cryodevice which can increase further cooling/warming rate than cryotop must be invented. Achieving optimum rate for oocytes (because of the small surface-to-volume ratio) and embryo (because of the large surface area and low water permeability) is the subject of numerous researches (46). 

It is good to mention draw attention to equilibration issue which is often critical in the case of oocytes cryopreserving. Since the oocyte is a large cell containing a large quantity of water, it requires time to reach adequate dehydration (osmotically balanced by the CPA solution) before lowering the temperature and thus it is more difficult to avoid ice crystal formation. The pretreatment (or equilibration) time before cooling might affect the viability and developmental ability of oocytes (18). Comparing the SRs of oocytes pretreated to 0.75 M (Exp. 4) with those exposed to 0.375 M CPAs (Exp. 5) lays emphasis on the vital effect of pretreatment in terms of CPA-concentration, exposure temperature and duration. 

Any intervention causing even temporary change in the equilibrium of the physiological state could potentially be toxic to cells, including ICSI, which was introduced as a unique technique to overcome cryo-induced zona hardening and sequential IVF failure (5). Due to mechanical stresses and small amounts of PVP into oocytes during procedure (9), the movement of the polar body in the perivitelline space or migration of the spindle deeper into the oocyte could be misleading as for the relation between the spindle and polar body (47), so that ICSI technique has fallen into harmful category. These can be the reason why none of the cryosurvived oocytes in Exp. 4 were fertilized. In addition to the immediate causes of the cryodamage explained above, we shall make reference to the work of Tucker and Libermann (16). who have provided evidence for the fact that although the cell nucleus has the ability to reassemble morphologically following cryopreservation, the future development of the embryo could be suboptimal. Spindle and chromatin re-organization may not be the whole point but it is a vital component in any dividing cells nucleus. The degree of CSK repolymerization was critically dependant on the method used to dilute or remove the CPAs (48). Post-warming incubation period brings about reassembled spindles in vitrified oocytes. Furthermore, controversy continues over the striking resemblance of the reassembled spindle with the original one (49). In order to guarantee the used protocol, one needs to find the answer of the raised question how to carry out warming procedure to make useful contribution in preventing original spindle depolymerization and grasping an opportunity to have the original spindle throughout vitrification/ warming procedure. 

Except for the results published by Schroeder *et al *who were able to successfully cryopreserve mouse oocytes using a slow-cooling protocol and 1.0 M DMSO (50), this appears to be the first time that 1.0 M and 0.75 M concentrated CPAs + 0.5 M sucrose have been evaluated for cryotop-vitrified mouse M-II oocytes (Exp. 3 and 4). Experience led us to the expectation that in the course of cryopreservation with 1.0 M concentrated CPAs for oocytes freezing, the oocytes viability will be reduced, although not necessarily to an extent that makes them incapable of becoming fertilized or developed further. It is suggested that the current reduced concentrated solutions i) could be combined with other additives (such as CSK stabilizers (51), ice blocking polymers (52) and high concentration of the sugars (53) into, or ii) to deplete some of the supplements from freezing solutions [such as sodium (54-55) and Ca^2+^ ions (56)], in such circumstances with iii) different pretreatment temperature, duration and the concentration of the CPAs (45). And finally, we draw your attention to a large body of experimental evidences that indicate major positive impacts of cumulus cells on vitality of oocytes (51, 57-59). It would be effective to study the efficiency of the new protocol on cryopreserving cumulus oocyte complex; furthermore, because of the mechanical stress of PZD/ICSI procedures, monitoring oocyte parthenogenetic activity (18) or adopting conventional IVF following PZD are safer alternatives to scoring oocyte functioning after warming. In turn, it may lead to some improvements in cryopreservation unfertilized oocytes procedures. 

## Conclusion

From the results of this study it can be concluded that vitrification by cryotop technology using minimal volume approach increases both cooling and warming rates. Therefore, the CPAs (EG+DMSO) limited reduction to 1.25 and 1.0 M instead of 1.5 M for oocytes and embryos cryotop-vitrification procedure, which may be a slight adjustment.

## References

[1] Tucker M, Morton P, Liebermann J (2004). Human oocyte cryopreservation: a valid alternative to embryo cryopreservation?. Obstet Gynecol.

[2] Kim ChG, Yong H, Lee G, Cho J (2008). Effect of the polyvinylpyrrolidone concentration of cryoprotectant on mouse embryo development and production of pups: 7.5% of PVP is beneficial for In Vitro and In Vivo development of frozen-thawed mouse embryos. J Reprod Dev.

[3] Sheehan CB, Lane M, Gardner DK (2006). The cryoloop facilitates re-vitrification of embryos at four successive stages of development without impairing embryo growth. Hum Reprod.

[4] Ciotti PM, Porcu E, Notarangelo L, Magrini O, Bazzocchi A, Venturoli S (2008). Meiotic spindle recovery is faster in vitrification of human oocytes compared to slow freezing. Fertil Steril.

[5] Elder K, Dale B (2003). In vitro fertilization.

[6] Makarevich AV, Chrenek P, Olexikova L, Popelkova M, Turanova Z, Ostro A (2008). Post-thaw survival, cell death and actin cytoskeleton in gene-microinjected rabbit embryos after vitrification. Theriogenology.

[7] Almasi-turk S, Roozbehi A, Aliabadi E, Sadeghi Y, Haeri A, Hosseini A (2009). Developmental consequences of mouse cryotop-vitrified oocyte and embryo using low concentrated CPAs. Iran J Reprod Med.

[8] Eroglu A, Lawitts JA, Toner M, Toth TL (2003). Quantitative microinjection of trehalose into mouse oocytes and zygotes, and its effect on development. Cryobiology.

[9] Eroglu A, Bailey SE, Toner M, Toth TL (2009). Successful cryopreservation of mouse oocytes by using low concentrations of trehalose and dimethylsulfoxide. Biol Reprod.

[10] Kyono K, Nakajo Y, Kumagai S, Nishinaka C, Tucker MJ, Liebermann J (2007). Vitrifying and warming of oocytes using cryotop. Vitrification in assisted reproduction: A user’s manual and trouble shooting guide.

[11] Soleimani R, Van der Elst J, Heytens E, Van den Broecke R, Gerris J, Dhont M (2008). Back muscle as a promising site for ovarian tissue transplantation, an animal model. Hum Reprod.

[12] Balaban B, Urman B, Alatas C, Mercan R, Mumcu A, Isiklar A (2002). A comparison of four different techniques of assisted hatching. Hum Reprod.

[13] Zenzes MT, Bielecki R, casper RF, Leibo SP (2001). Effects of chilling to 0°C on the morphology of meiotic spindles in human metaphase II oocytes. Fertil Steril.

[14] Kuwayama M (2007). Highly efficient vitrification for cryopreservation of human oocytes and embryos: The Cryotop method. Theriogenology.

[15] Gardner DK, Weissman A, Howless CM, Shoham Z (2004). Text book of assisted reproductive techniques: Laboratory and clinical perspectives.

[16] Tucker MJ, Liebermann J (2007). Vitrification in assisted reproduction: A user’s manual and trouble shooting guide.

[17] Yamada C, Caetano HVA, Simoes R, Nicacio AC, Feitosa WB, Assumpcao MEOD (2007). Improved bovine oocyte cryopreservation: Comparison of different associations with ethylene glycol, glycerol and dimethylsulfoxide. Anim Reprod Sci.

[18] Endoh K, Mochida K, Ogonuki N, Ohkawa M, Shinmen A, Ito M (2007). The developmental ability of vitrified oocytes from different mouse strains assessed by parthenogenetic activation and intaracytoplasmic sperm injection. J Reprod Dev.

[19] Kartberg AJ, Hambiliki F, Arvidsson T, Stravreus-Evers A, Svalander P (2008). Vitrification with DMSO protects embryo membrane integrity better than solutions without DMSO. Reprod Biomed Online.

[20] Manjunatha BM, Gupta PSP, Ravindra JP, Devaraj M, Nandi S (2009). Effect of vitrification medium composition and exposure time on post-thaw development of buffalo embryos produced in vitro. Vet J.

[21] Cobo A, Kuwayamam M, Perezm S, Ruiz A, Pellicer A, Remohi J (2008). Comparison of concomitant outcome achieved with fresh and cryopreserved donor oocytes vitrified by the cryotop method. Fertil Steril.

[22] Kuwayama M, Vajta G, Kato O, Leibo SP (2005). Highly efficient vitrification method for cryopreservation of human oocytes. Reprod Biomed Online.

[23] Katayama KP, Stehlik J, Kuwayama M, Kato O, Stehlik E (2003). High survival rate of vitrified human oocytes results in clinical pregnancy. Fertil Steril.

[24] Morato R, Izquierdo D, Paramio MT, Mogas T (2008). Cryotops versus open-pulled straws (OPS) as carriers for the cryopreservation of bovine oocytes: Effects on spindle and chromosome configuration and embryo development. Cryobiology.

[25] Songsasen N, Yu IIJ, Ratterree MS, Vandevoort CA, Leibo SP (2002). Effect of chilling on the organization of tubulin and chromosomes in rhesus monkey oocytes. Fertil Steril.

[26] Mandelbaum J, Anastasiou O, Levy R, Guerin JF, Larouziere V, Antoine JM (2004). Effects of cryopreservation on the meiotic spindle of human oocytes. Obstet Gynecol.

[27] Nagai S, Mabuchi T, Hirata S, Shoda T, Kasai T, Yokota S (2004). Correlation of abnormal mitochondria distribution in mouse oocytes with reduced developmental competence. Tohoku J Exp Med.

[28] Bettencourt EMV, Bettencourt CM, Silva JNCE, Ferreira P, Matos CP, Oliveira E (2009). Ultrastructural characterization of fresh and cryopreservaed in vivo produced ovine embryos. Theriogenology.

[29] Bromfield JJ, Coticchio G, Hutt K, Sciajno R, Borini A, Albertini DF (2009). Meiotic spindle dynamics in human oocytes following slow-cooling cryopreservation. Hum Reprod.

[30] Zhou GB, Li N (2009). Cryopresrvation of porcine oocytes: recent advances. Mol Hum Reprod.

[31] Mullen SF, Rosenbaum M, Critser JK (2007). The effect of osmotic stress on the cell volume, metaphase II spindle and developmental potential of in vitro matured porcine oocytes. Cryobiology.

[32] Shi WQ, Zhu SE, Zhang D, Wang WH, Tang GL, Hou YP (2006). Improved development by taxol pretreatment after vitrification of in vitro matured porcine oocytes. Reproduction.

[33] Tharasanit T, Colenbrander B, Stout TAE (2005). Effect of cryopreservation on the cellular integrity of equine embryos. Reprod Online.

[34] Kim NH, Chung KS, Day N (1997). The distribution and requirements of microtubules and microfilaments during fertilization and parthenogenesis in pig oocytes. J Reprod Fertil.

[35] Paynter SJ (2000). Current status of the cryopreservation of human unfertilized oocytes. Hum Reprod Update.

[36] Isachenko EF, Nayudu PL (1997). Vitrification of mouse germinal vesicle oocytes: effect of treatment temperature and egg yolk on chromatin and spindle normality and cumulus integrity. Hum Reprod.

[37] Katayama M Zhongm Zh, Lai L, Sutovsky P, Prather RS, Schatten H (2006). Mitochondrial distribution and microtubule organization in fertilized and cloned porcine embryos: implications for developmental potential. Dev Biol.

[38] Vajta G, Kuwayama M, Vanderzwalmen P, MJ Tucker, J Libermann J (2007). Disadvantages and benefits of vitrification. Vitrification in assisted reproduction.

[39] Braga DPAF, Figueira RCS, Rodrigues D, Madaschi C, Pasqualotto FF, Iaconelli A, Borges E (2008). Prognostic value of meiotic spindle imaging on fertilization rate and embryo development in in vitro-matured human oocytes. Fertil Steril.

[40] Lowther KM, Weitzman VN, Maier D, Mehlmann LM (2009). Maturation, fertilization, and the structure and function of the endoplasmic reticulum in cryopreserved mouse oocytes. Biol Reprod.

[41] Gomes CM, Silva CASE, Acevedo N, Baracat E, Serafini P, Smith GD (2008). Influence of vitirification on mouse metaphase II oocyte spindle dynamics and chromatin alignment. Fertil Steril.

[42] Amir A, Zvi R (2008). Do chilling injury and heat stress share the same mechanism of injury in oocytes?. MCE.

[43] Stachecki JJ, Cohen J, Schimmel T, Willadson SM (2002). Fetal development of mouse oocytes and zygotes cryopreserved in a nonconventional freezing medium. Cryobiology.

[44] Arav A, Yavin S (2007). Measurement of essential physical properties of vitrification solutions. Theriogenology.

[45] Pegg DE, JG Day, GN Stacey (2006). Principles of Cryopreservation. Cryopreservation and freeze-drying protocols. Methods in molecular biology.

[46] Gardner DK, Sheehan CB, Rienzi L, Katz-jaffe M, Larman MG (2007). Analysis of oocyte physiology to improve cryopreservation procedures. Theriogenology.

[47] Wang WH, Meng L, Hackett RJ, Odenbourg R, Keefe DL (2001). The spindle observation and its relationship with fertilization after intracytoplasmic sperm injection in living human oocytes. Fertil Steril.

[48] Skidmore JA, Schovers E, Stout TAE (2008). Effect of different methods of cryopreservation on the cytoskeletal integrity of dromedary camel (Camelus dromedaritus) embryos. Anim Reprod Sci.

[49] Rienzi L, Martinez F, Ubaldi F, Minasi MG, Lacobelli M, tesarik J (2004). Polscope analysis of meiotic spindle changes in living metaphase II human oocytes during the freezing and thawing procedures. Hum Reprod.

[50] Schroeder AC, Champlin AK, Mobraaten LE, Eppig JJ (1990). Developmental capacity of mouse oocytes cryopreserved before and after maturation in vitro. J Reprod Fertil.

[51] Zhang J, Nedambale TL, Yang M, Li J (2009). Improved development of ovine matured oocyte following solid surface vitrification (SSV): Effect of cumulus cells and cytoskeleton stabilizer. Anim Reprod Sci.

[52] Wang HY, Lu SS, Lun ZR (2009). Glass transition behavior of the vitrification solutions containing propanediol, dimethyl sulfoxide and polyvinyl alcohol. Cryobiology.

[53] Eroglu A, Toner M, Toth TL (2002). Beneficial effect of microinjected trehalose on the cryosurvival of human oocytes. Fertil Steril.

[54] Stachecki JJ, Cohen J, Willadsoen S (1998). Cryopreservation of unfertilized mouse oocytes: The effect of replacing sodium with choline in the freezing medium. Cryobiology.

[55] Stachecki JJ, Cohen J, Willadsen S (1998). Detrimental effects of sodium during mouse oocyte cryopreservation. Biol Rerod.

[56] Larman MK, Sheehan CB, Gardner DK (2006). Calcium-free vitrification reduces cryoprotectant-induced zona pellucida hardening and increases fertilization rates in mouse oocytes. Reproduction.

[57] Chian RC, Kuwayama M, Tan L, Tan J, Kato O, Nagai T (2004). High survival rate of bovine oocytes matured in vitro following vitrification. J Reprod Dev.

[58] Rupert-Lingham CJ, Paynter SJ, Godfrey J, Fuller BJ, Shaw RW (2003). Developmental potential of murine germinal vesicle stage cumulus-oocyte complexes following exposure to dimethylsulphoxide or cryopreservation: Loss of membrane integrity of cumulus cells after thawing. Hum Reprod.

[59] VandeVoort CA, Shirley CR, Hill DL, Leibo SP (2008). Effects of CPAs and cryopreservation on germinal vesicle-stage cumulus-oocyte complexes of rhesus monkeys. Fertil Steril.

